# Global identification of circular RNAs in imatinib (IM) resistance of chronic myeloid leukemia (CML) by modulating signaling pathways of circ_0080145/miR-203/ABL1 and circ 0051886/miR-637/ABL1

**DOI:** 10.1186/s10020-021-00395-z

**Published:** 2021-11-15

**Authors:** Yao-hua Lu, Zhong-yi Huang

**Affiliations:** 1 Present Address: Department of Pharmacy, Sixth People’s Hospital Affiliated to Shanghai Jiaotong University, Shanghai, 200233 China; 2 Department of Pharmacy, Jing’an District Central Hospital, No 259 Xikang Road, Jing’an District, Shanghai, 200040 China

**Keywords:** Chronic myeloid leukemia, Circular RNAs, ABL1, Imatinib resistance, miRNA

## Abstract

Imatinib (IM), targeting of BCR-ABL1 tyrosine kinase, is currently one of the first-line choices in the treatment of chronic myeloid leukemia (CML). This study aims to explore the molecular mechanisms underlying IM resistance in CML treatment. 108 CML patients were recruited and grouped according to their sensitivity to IM as the responder group (N = 66) and the non-responder group (N = 42). Real-time quantitative PCR (RT-qPCR) was performed to evaluate the expression of candidate circular RNAs (circRNAs), microRNA (miRNAs) and messenger RNA (mRNAs). No significant difference was noted regarding demographic and clinicopathological characteristics between the responder group and the non-responder group. The expression of circ_0080145, circ_0051886 and ABL1 mRNA was significantly increased, while the expression of miR-203 and miR-637 was decreased in the non-responder group as compared with the responders. By using in-silicon analysis, it was predicted that circ_0080145 and circ_0051886 targeted miR-203 and miR-637 respectively, and ABL1 was found to be shared direct target gene of miR-203 and miR-637. Ectopic over-expression of circ_0080145 and circ_0051886 respectively reduced the expression of miR-203 and miR-637. The expression of ABL1 mRNA/protein was most upregulated in culture cells co-transfected with circ_0080145 and circ_0051886 as compared with those cells individually transfected. This study established the signaling pathways of circ_0080145/miR-203/ABL1 and circ 0051886/miR-637/ABL1. The deregulation of circ_0080145 and circ_0051886 is, at least partially, responsible for the development of IM chemoresistance in CML by regulating expression of ABL1 via modulating expression of miR-203 and miR-637.

## Introduction

Chronic myeloid leukemia (CML) is featured by the chromosomal translocation of 22q1 with 9q34 to produce a BCR/ABL1 fusion gene (Machova Polakova et al. 2013; Naka et al. 2015). Meanwhile, the resistance to chemotherapy continues to be a significant problem in the treatment of CML (Baccarani et al. 2009). As a result, it is important to clarify the molecular pathways underlying the development of IM chemoresistance in CML to improve CML treatment as well as prognosis.

Originally, circular RNAs (circRNAs) were considered as a product of error in RNA splicing. However, the latest researches showed that circRNAs were involved in tumorigenesis by regulating expression of genes or other non-coding RNAs such as microRNAs (miRNAs) (Bonizzato et al. 2016). Furthermore, circRNAs including circ_0080145 (Liu et al. 2018), circHIPK3 (Feng et al. 2020), circ_BA9.3 (Pan et al. 2018) and circ_100053 (Ping et al. 2019) have been reported to be involved in the pathogenesis of chronic myeloid leukemia. MiRNA is another cluster of non-coding RNA and it has also been reported to be deregulated in several kinds of cancers such as head and neck carcinoma, laryngeal squamous cell cancer, as well as non-small cell lung carcinoma. For instance, miR-203 has been reported to regulate a variety of biological processes including proliferation, invasion and apoptosis (Tian et al. 2014; Obayashi et al. 2016; Hu et al. 2016; He et al. 2017; Zheng et al. 2015; Viticchie et al. 2012). Furthermore, miR-637 was initially found in hepatocellular carcinoma to act as a tumor suppressor, and it seemed to play its anti-cancer role by disrupting the transcription of certain oncogenes (Slaby et al. 2009).

Imatinib (IM), a competitive tyrosine kinase inhibitor for ABL kinase, has been reported to significantly improve the overall survival of CML patients (Lucas et al. 2008). Although IM has been used as the first-line treatment for CML patients, there were still around 20–25% of the patients who did not achieve optimal response due to IM resistance (Lucas et al. 2008; Aloisi et al. 2006). And only a few patients achieved sustained molecular remission (Hochhaus et al. 2002). Factors including BCR-ABL point mutations and efflux transporter upregulation are associated with IM resistance (Aloisi et al. 2006; San José-Enériz et al. 2009; Flamant et al. 2010).

The t(9; 22) chromosomal translocation causes the constitutive activation of a stable tyrosine kinase BCR/ABL, which suppresses apoptosis while inducing the malignant transformation of CML (Stagno et al. 2016). Nevertheless, IM resistance may easily emerge because of BCR/ABL amplification or point mutations as reported in some cell lines (Gorre et al. 2001). Although many forms of BCR/ABL mutations are still sensitive to the second generation of tyrosine kinase inhibitors (TKIs) such as dasatinib or nilotinib, the T315I mutation of BCR/ABL remains refractory to the treatment by IM of any generation (Shah et al. 2004). For that reason, the study on alternative approaches in CML treatment is still of clinically significance. Even though various other mechanisms were speculated to cause IM resistance in patients with CML, although none of these mechanisms has been considered as a dominant source of IM resistance (Nasser et al. 2021; Bixby and Talpaz 2011; Bandres et al. 2009; Bitarte et al. 2011). Among those different factors causing IM resistance in CML patients, it was proposed that the differential expression of non-coding RNAs such as circRNAs, lncRNAs and miRNAs and specific genes may be a key factor of IM resistance (Gorre et al. 2001). For instance, several circRNAs including circ_0080145, circHIPK3, circ_100053, circ_BA9.3 and circ_0009910 have been reported to be involved in the pathogenesis of CML chemoresistance (Liu et al. 2018; Feng et al. 2020; Pan et al. 2018; Cao et al. 2020; Joshi et al. 2014; Li et al. 2013; Hershkovitz-Rokah et al. 2015). In this study, we first selected several candidate circRNAs based on microarray data from previous literature (Liu et al. 2018), and then we compared the expression of these selected circRNAS in the samples collected from the CML patients. Subsequently, we predicted candidate miRNAs that may interact with those selected circRNAs by using computational analysis (https://circinteractome.nia.nih.gov/index.html). Subsequently, we studied the potential interactions between the candidate miRNAs and ABL based on previous literature (Li et al. 2013) and online miRNA database (www.mirdb.org) to explore the molecular signaling pathways involved in IM resistance in CML.

## Materials and methods

### Patient recruitment

In this study, a total of 108 CML patients treated with IM were recruited and none of the patients carried any BCR/ABL1 mutations. Subsequently, according to their sensitivity to IM, the patients were grouped into a responder group (N = 66) and a non-responder group (N = 42). The definition of response to IM treatment was described previously (Chandrasekhar et al. 2019). The CML cancer cells were isolated using a method described previously (Järås et al. 2010). The treatment duration was 12 months for each participant. The demographic and clinicopathological characteristics of all patients in the two groups were collected and compared using Chi-square test. In this study, peripheral blood samples and CD34^+^ progenitor cells were harvested at the time of treatment. The age range of all patients included in this study was 20 to 63 years old. All experimental procedures were conducted based on the protocol approved by the Institutional Ethics Committee and the Declaration of Helsinki. Written forms of informed consent were received from all participants before the study was started.

### Cell culture and transfection

K562 cells (ATCC, Manassas, VA, US) were cultured in an RPMI 1640 medium (Gibco, Thermo Fisher Scientific, Waltham, MA) added with 10% of fetal bovine serum (FBS, Gibco, Thermo Fisher Scientific, Waltham, MA), 100 units per mL of penicillin, as well as 100 µg/mL of streptomycin. In this study, K562/R cells were generated by subjecting K562 cells to the exposure of IM in concentrations that gradually increased from 0.25 to 5 µM. After successful establishment, K562/R cells were cultured in RPMI 1640 added with 5 µM of IM. All cells used in this study were maintained in a 5% CO_2_ incubator at 37 °C and saturated humidity. When the cells reached 90% confluence, they were sub-cultured in a 1:4 ratio every 3 days. When the passage number of the cells reached 5, the cells were divided into different cell models, as shown below.

In cell treatment group 1, K562 cells, which were sensitive to IM, were induced with IM as shown above to generate IM resistant K562/R cells. Then, two groups of cells were set up: (1) K562 group; (2) K562/R group. The cells were cultured for 48 h and harvested to analyze the expression of target genes. In cell treatment group 2, K562 and K562/R cells were randomly divided into five groups, i.e., (1) K562 group (IM sensitive K562 cells); (2) K562/R + NC siRNA group (IM resistant K562 cells transfected with a negative control siRNA); (3) K562/R + circ_0080145 siRNA group (IM resistant K562 cells transfected with circ_0080145 siRNA); (4) K562/R + circ_0051886 siRNA group (IM resistant K562 cells transfected with circ_0051886 siRNA); (5) K562/R + circ_0080145 siRNA + circ_0051886 siRNA group (IM resistant K562 cells transfected with both circ_0080145 siRNA and circ_0051886 siRNA). In cell treatment group 3, K562 and K562/R cells were randomly divided into four groups, i.e., (1) K562 group (IM sensitive K562 cells); (2) K562 + p-circ_0080145 group (IM sensitive K562 cells transfected with a plasmid carrying circ_0080145); (3) K562 + p-circ_0051886 group (IM sensitive K562 cells transfected with a plasmid carrying circ 0051886); (4) K562 + p-circ_0080145 + p-circ_0051886 group (IM sensitive K562 cells transfected with both plasmids carrying circ_0051886 and circ_0080145). All transfection experiments were carried out using Lipofectamine^2000^ (Invitrogen, Carlsbad, CA) following the manufacture’s protocol. The cells were harvested at 48 h post-transfection to analyze the expression of target genes.

### RNA isolation and real-time PCR

Real time PCR was carried out to determine the relative expression of circ_0051886, circ_0080145, miR-637, miR-203 and ABL1 mRNA in each sample. In brief, after total RNA was extracted using a Trizol reagent (Invitrogen, Carlsbad, CA) following the protocol recommended by the reagent manufacturer. The extracted RNA was reverse transcribed into cDNA using a Taqman Reverse Transcription assay kit (Thermo Fisher Scientific, Waltham, MA) following the protocol recommended by the assay kit manufacturer. In the next step, real time PCR was carried out utilizing an SYBR Green Real-time master mixes kit (Thermo Fisher Scientific, Waltham, MA) on a PRISM 7500 real time PCR machine (Applied Biosystems, Foster City, CA) following the protocol recommended by the assay kit manufacturer. Finally, the relative expression of circ 0051886, circ_0080145, miR-637, miR-203 and ABL1 mRNA in each sample was calculated by using the 2^−ΔΔCT^ threshold cycle approach (Livak and Schmittgen 2001). Also, the relative expression of BCR/ABL1 was measured by PCR following previously published methods (Chandrasekhar et al. 2019; Beillard et al. 2003).

### Vector construction, mutagenesis, and luciferase assay

By using the bioinformatic tool (https://circinteractome.nia.nih.gov/index.html), we found a potential circ_0080145 binding sites located on miR-203, as well as a putative circ_0051886 binding sites on miR-637. To verify the regulatory relationship between miR-203 and circ_0080145, as well as between miR-637 and circ 0051886, we generated wild type vectors of miR-203 and miR-637, respectively, by inserting their sequences containing the circ_0080145 and circ_0051886 binding sites into pcDNA vectors (Promega, Madison, WI). At the same time, we generated site-directed mutations in the circ_0080145 and circ_0051886 binding sites of miR-203 and miR-637, respectively, using a Quick Change II mutagenesis assay kit (Stratagene, San Diego, CA) following the protocol recommended by the assay kit manufacturer. Then, miR-203 and miR-637 sequences were also inserted into pcDNA vectors to generate mutant-type vectors of miR-203 and miR-637, respectively. In the next step, K562 cells were co-transfected with wild type/mutant type vectors of miR-203 and miR-637 in conjunction with circ_0080145 and circ 0051886, respectively, using Lipofectamine 2000 (Invitrogen, Carlsbad, CA) following the protocol recommended by the reagent manufacturer. At 48 h post-transfection, the luciferase activity of transfected cells was measured by using a Bright-Glo luciferase assay (Promega, Madison, WI) and a TD-20/20 luminometer. Similarly, by using the bioinformatic tool (www.mirdb.org), the putative binding sites of miR-203 and miR-637, respectively, were observed on the 3ʹUTR of ABL1 mRNA. To verify the regulatory relationship between miR-203/miR-637 and ABL1 mRNA, we also generated wild-type and mutant vectors of ABL1 mRNA carrying miR-203 and miR-637 binding sites, and the vectors were also respectively transfected into K562 cells along with miR-203 and miR-637. Then, the luciferase activity of transfected cells was measured at 48 h post-transfection.

### Western blot analysis

To determine the protein expression of BCR/ABL1 in collected samples, the samples were first treated with a RIPA lysis buffer (Sangon, Shanghai, China) following the protocol recommended by the reagent manufacturer to obtain total protein, which was then resolved by 10% SDS-PAGE. In the next step, the resolved proteins were electroblotted onto PVDF membranes, which were then blocked for 1 h at room temperature with TBST containing 5% skim milk, and subsequently incubated with primary anti-BCR/anti-ABL1 antibodies and then suitable HRP-labeled secondary antibodies according to the incubation conditions suggested by the antibody manufacturer (Abcam, Cambridge, MA). Finally, after development using an enhanced chemiluminescence reagent (Thermo Fisher Scientific, Waltham, MA), the protein bands were analyzed by using a chemiluminescence imaging system to calculate the relative expression of BCR and ABL1 proteins in each sample.

### MTT assay

The status of cell proliferation was measured by using a standard MTT assay (Thermo Fisher Scientific, Waltham, MA) following the protocol recommended by the reagent manufacturer.

### Apoptosis assay with flow cytometry

The status of cell apoptosis was measured by using an Alexa Fluor 488 Annexin V/Dead Cell Apoptosis assay kit (Thermo Fisher Scientific, Waltham, MA) following the protocol recommended by the reagent manufacturer.

### Statistical analysis

All statistical analyses were carried out by using Prism 6.0 software (GraphPad, San Diego, CA). Student’s t-tests were used to compare the differences among two different groups, while one-way ANOVA and Tukey’s test (as post hoc test) were used to compare the differences among multiple groups. P < 0.05 was considered to be statistically significant differences. Data were presented as mean ± SD.

## Results

### Comparison of expression of candidate circRNAs, ABL1 mRNA and BCR/ABL1 between responders and non-responders in CML

**Fig. 1:**
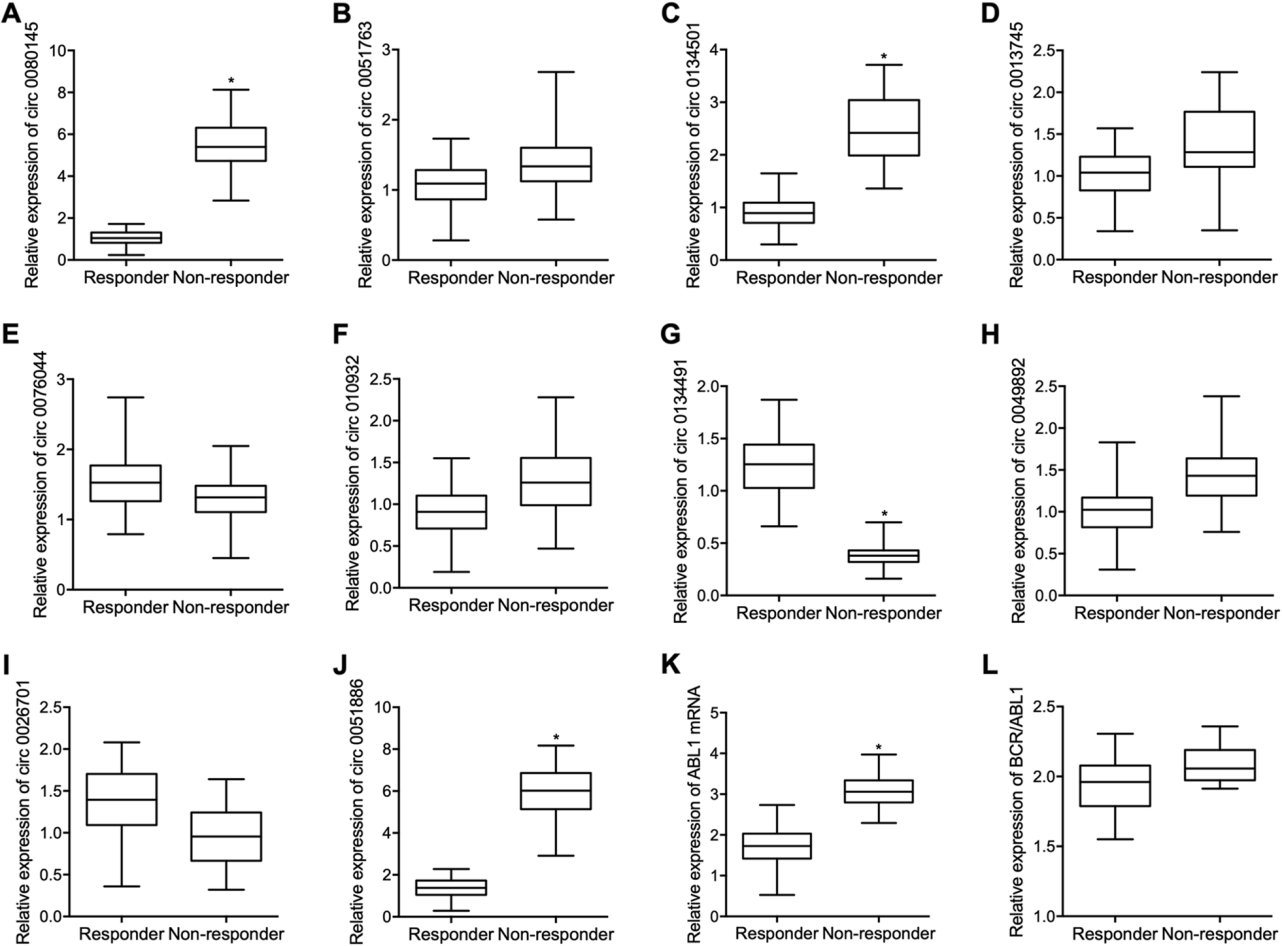
Among all candidate circRNAs, circ_0080145, circ 0134501, circ 0134491 and circ_0051886 were dysregulated between different CML patient groups, and unlike BCR/ABL1, the level of ABL1 mRNA was higher non-responder patients (*P value < 0.05 vs. responder group). **A** The relative expression level of circ_0080145 was higher in CML patients sensitive to IM; **B** The relative expression levels of circ 0051763 were similar in different CML patient groups; **C** The elative expression level of circ 0134501 was higher in CML patients sensitive to IM; **D** The relative expression levels of circ 0013745 were similar in different CML patient groups; **E** The relative expression levels of circ 0076044 were similar in different CML patient groups; **F** The relative expression levels of circ 0109320 were similar in different CML patient groups; **G** The relative expression level of circ 0134491 was lower in CML patients sensitive to IM; **H** The relative expression levels of circ 0049892 were similar in different CML patient groups; **I** The relative expression levels of circ 0026701 were similar in different CML patient groups; **J** The relative expression level of circ_0051886 was higher in CML patients sensitive to IM; **K** The relative expression level of ABL1 mRNA was higher in CML patients sensitive to IM; **L** The relative expression levels of BCR/ABL1 were similar in different CML patient groups

As shown in Table 1, we compared the demographic and clinicopathological characteristics between the two CML patient groups. Accordingly, no significant difference was noted between the responders and the non-responders. As indicated by the RT-qPCR, among the 10 candidate circRNAs selected for the study of IM resistance in the treatment of CML, the expression levels of circ 0051763 (Fig. 1B), circ 0013745 (Fig. 1D), circ 0076044 (Fig. 1E), circ 0109320 (Fig. 1F), circ 0049892 (Fig. 1H) and circ 0026701 (Fig. 1I) were comparable between the responders and the non-responders. In addition, the expression of circ_0080145 (Fig. 1A), circ 0134501 (Fig. 1C) and circ_0051886 (Fig. 1J) was all significantly increased in the non-responders as compared with responders. On the contrary, circ 0134491 (Fig. 1G) was downregulated in the non-responders. Moreover, the relative expression of ABL1 mRNA was much higher in non-responders than responders (Fig. 1H), while the relative expression of BCR/ABL1 was similar among different CML patient groups (Fig. 1I).

**Table 1:** Demographic and clinical characteristics of CML patients sensitive and insensitive to IM treatment

Characteristics	Serum circHIPK3		X^2^ test *P* value
	Responder (N = 66)	Non-responder (N = 42)	
Age			
< 60	42	31	0.531
≥ 60	24	11	
Gender			
Male	36	25	0.339
Female	30	17	
Sokal RR			
Low	25	17	0.744
Intermediate	23	15	
High	18	10	
BCR/ABL			
Mutated	0	0	0.209
Unmutated	66	42

### Comparison of expression of candidate circRNAs in sensitive and resistant K562 cells

**Fig. 2:**
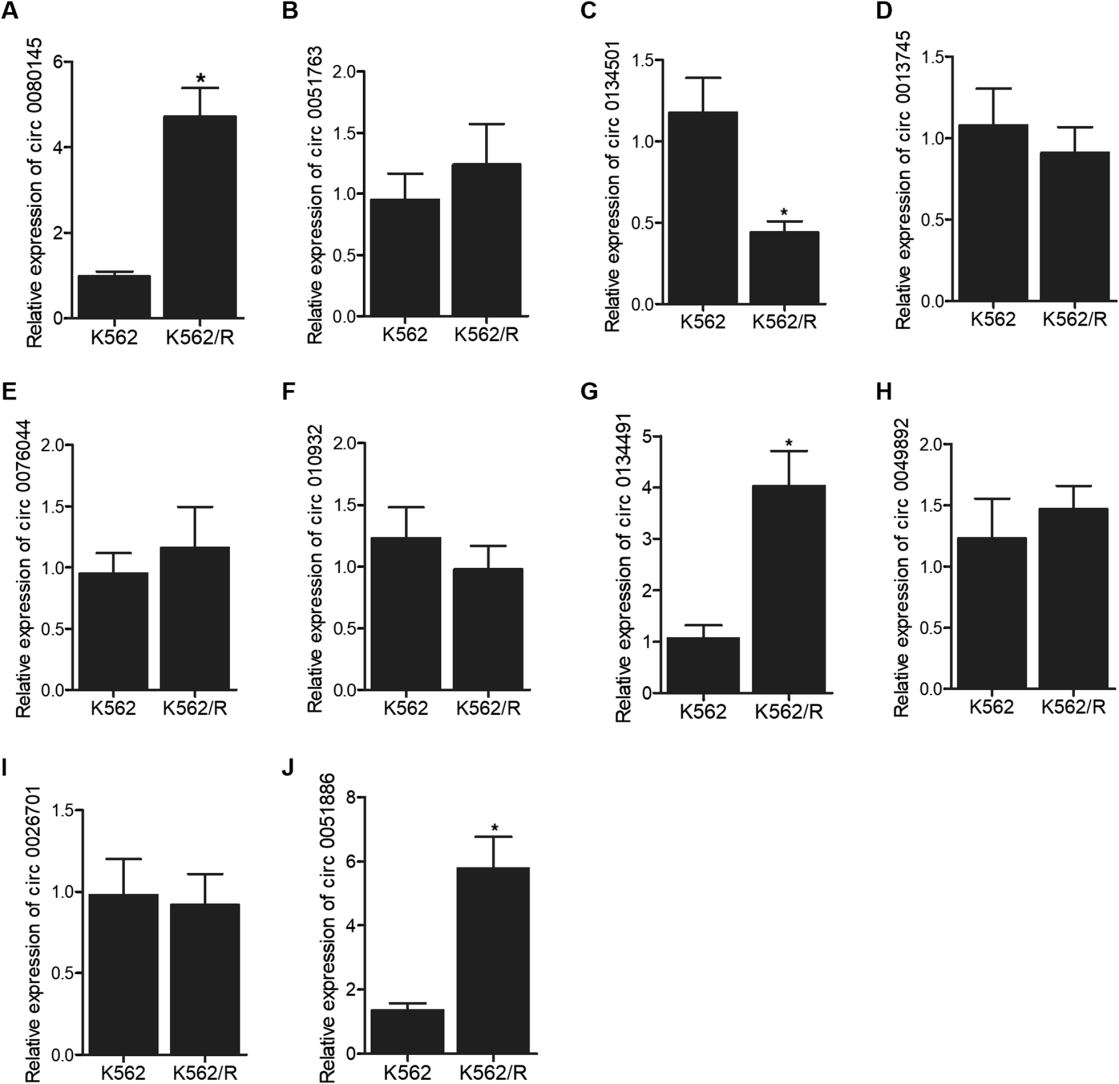
The expression of candidate circRNAs in K562 cell groups indicated that circ_0080145, circ 0134501, circ 0134491 and circ_0051886 were dysregulated in K562/R cells (*P value < 0.05 vs. K562 group). **A** The relative expression level of circ_0080145 was higher in K562/R cells; **B** The relative expression levels of circ 0051763 were similar in K562 and K562/R cell groups; **C** The relative expression level of circ 0134501 was lower in K562/R cells; **D** The relative expression levels of circ 0013745 were similar in K562 and K562/R cell groups; **E** The relative expression levels of circ 0076044 were similar in K562 and K562/R cell groups; **F** The relative expression levels of circ 0109320 were similar in K562 and K562/R cell groups; **G** The relative expression levels of circ 0134491 were higher in K562/R cells; **H** The relative expression levels of circ 0049892 were similar in K562 and K562/R cell groups; **I** The relative expression levels of circ 0026701 were similar in K562 and K562/R cell groups; **J** The relative expression levels of circ_0051886 were higher in K562/R cells

Resistant K562 cells were induced and established by being exposed to different concentration of IM as described in “Materials and methods” section: the parental K562 cells were sensitive to IM and the K562/R cells were resistant to IM. The qPCR results showed that the expression of circ_0080145 (Fig. 2A), circ 0134491 (Fig. 2G) and circ_0051886 (Fig. 2J) was upregulated in the K562/R group, while circ 0134501 (Fig. 2C) was down-regulated in the K562/R group compared with parental K562 cells. Meanwhile, the expression of circ 0051763 (Fig. 2B), circ 0013745 (Fig. 2D), circ 0076044 (Fig. 2E), circ 0109320 (Fig. 2F), circ 0049892 (Fig. 2H) and circ 0026701 (Fig. 2I) was comparable between the sensitive and resistant cells.

### Establishment of signaling pathways

**Fig. 3:**
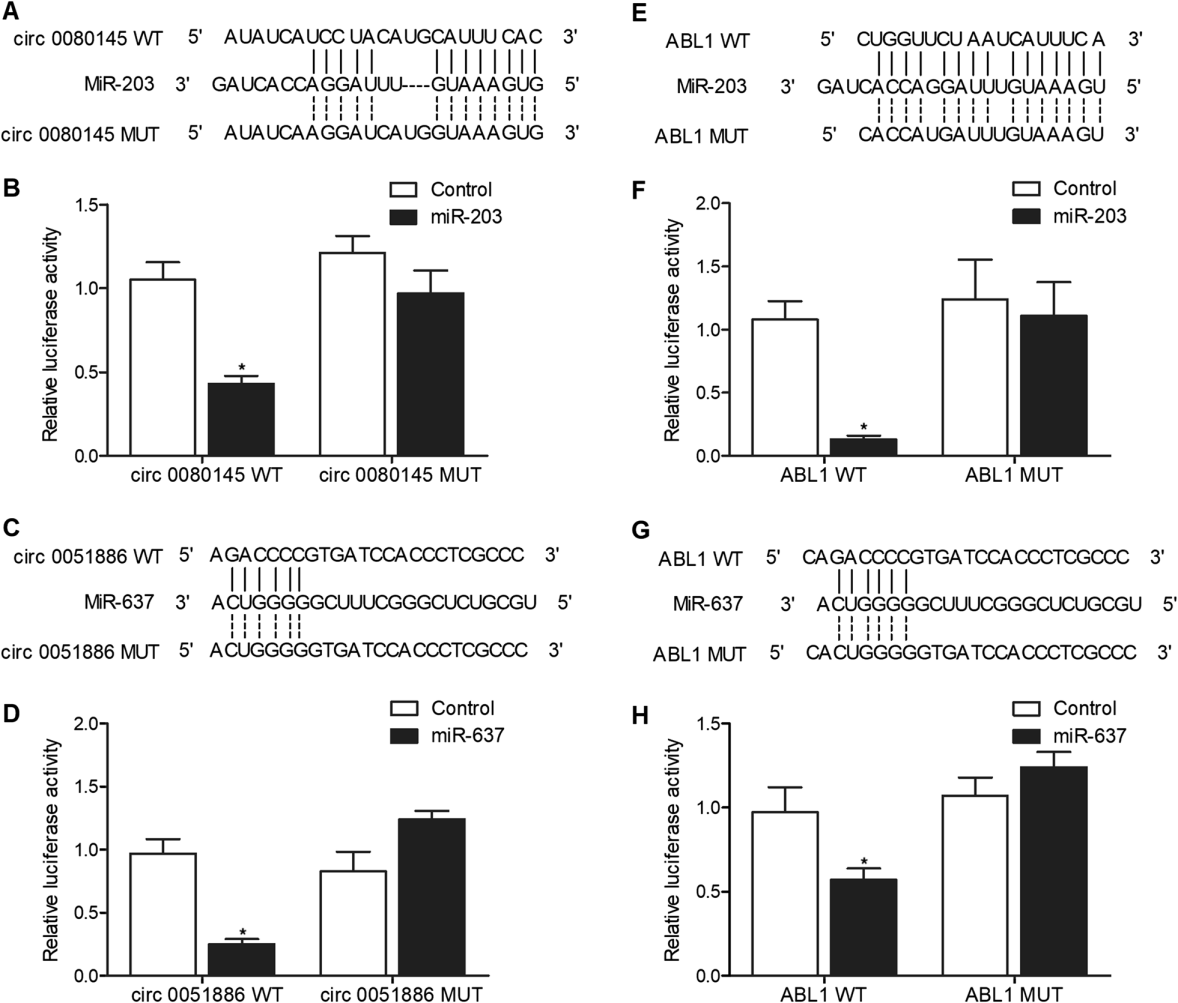
The molecular relationships between circ_0080145, miR-203 and ABL1, as well as circ 005188, miR-637 and ABL1, were established via sequence analysis and luciferase assays (*P value < 0.05 vs. Control group). **A** A potential binding site of miR-203 was found on circ_0080145; **B** Luciferase assay validated that circ_0080145 targeted miR-203; **C** A potential binding site of miR-637 was found on circ 0051886; **D** Luciferase assay validated that circ_0051886 targeted miR-637; **E** A putative binding site of miR-203 was observed on the 3ʹUTR of ABL1 mRNA; **F** Luciferase assay validated that ABL1 mRNA was a target of miR-203; **G** A putative binding site of miR-637 was observed on the 3ʹUTR of ABL1 mRNA; **H** Luciferase assay validated that ABL1 mRNA was a target of miR-637

By using the bioinformatic tool (https://circinteractome.nia.nih.gov/index.html), we predicted a potential binding site of miR-203 on circ_0080145 (Fig. 3A), and the relative luciferase activity (RLA) of circ_0080145 was reduced in K562 cells transfected with miR-203 (Fig. 3B), suggesting that circ_0080145 may sponge miR-203. A similar putative binding site miR-637 was also observed on circ_0051886 (Fig. 3C), and the reduced RLA of circ_0051886 in the presence of miR-637 also confirmed the interaction between circ_0051886 and miR-637 (Fig. 3D). Two putative binding sites were observed on the 3ʹUTR of ABL1 mRNA which respectively enable the bindings of ABL1 mRNA to miR-203 (Fig. 3E) and miR-637 (Fig. 3G). Accordingly, the RLA of ABL1 mRNA 3ʹUTR but not that of mutated ABL1 mRNA 3’UTR in K562 cells was evidently inhibited by the transfection of miR-203 (Fig. 3F) or miR-637 (Fig. 3H), thus validating that miR-203 and miR-637 could respectively target ABL1 mRNA. Therefore, two different signaling pathways, circ_0080145/miR-203/ABL1 and circ 0051886/miR-637/ABL1, were established.

### Comparison of expression of miR-203 and miR-637 between responders and non-responders

**Fig. 4:**
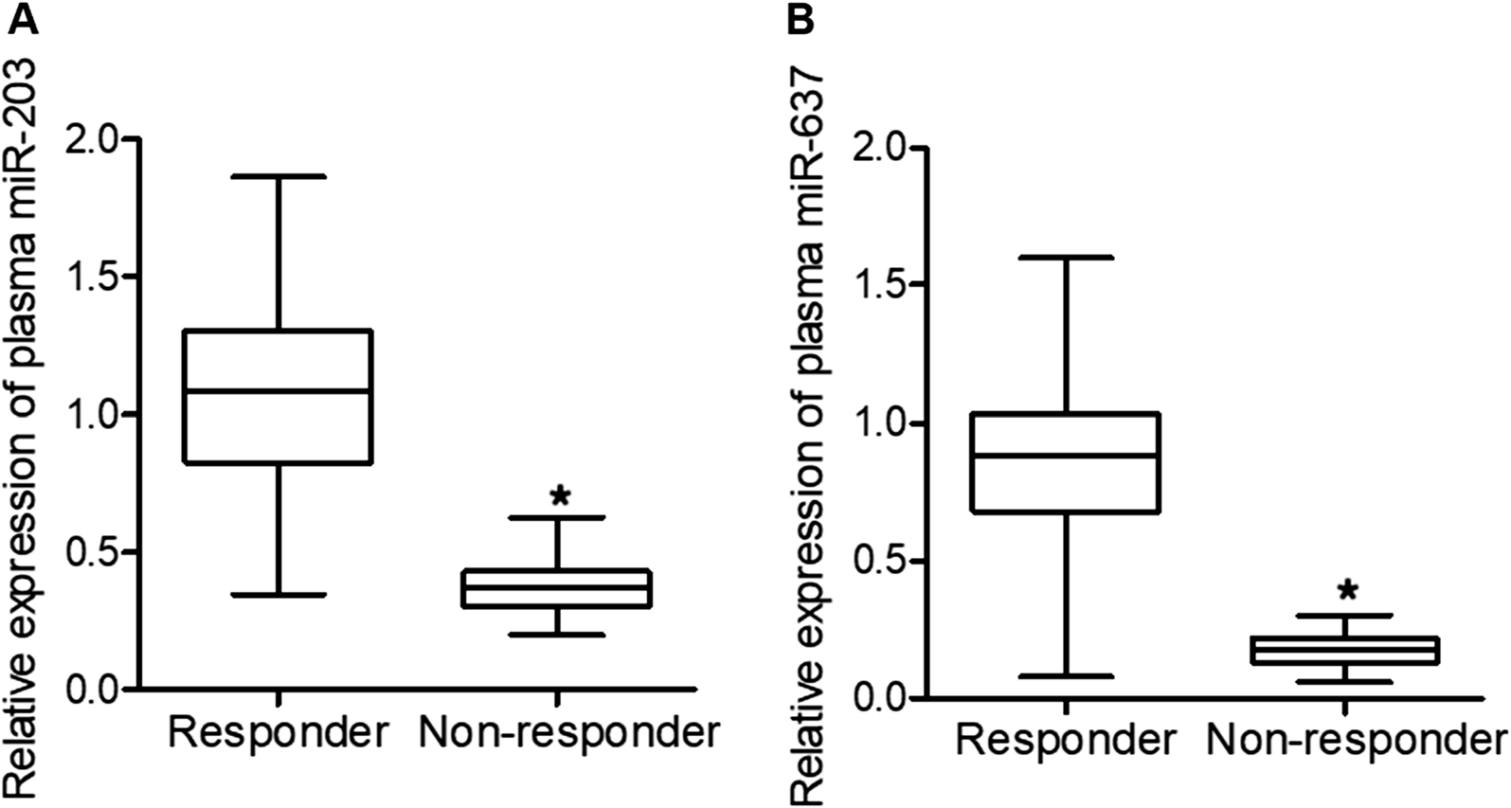
Expression of miR-203 and miR-637 were lower in non-responder patient groups (*P value < 0.05 vs. responder group). **A** The expression of miR-203 was down-regulated in CML patients sensitive to IM; **B** The expression of miR-637 was down-regulated in CML patients sensitive to IM

The expression of miR-203 and miR-637 was also measured in the two groups of CML patients and we found that the expression of miR-203 (Fig. 4A) and miR-637 (Fig. 4B) in CML patients sensitive to IM were both significantly downregulated compared with that in non-responders.

### Validation of the signaling pathways in K562 cells

**Fig. 5:**
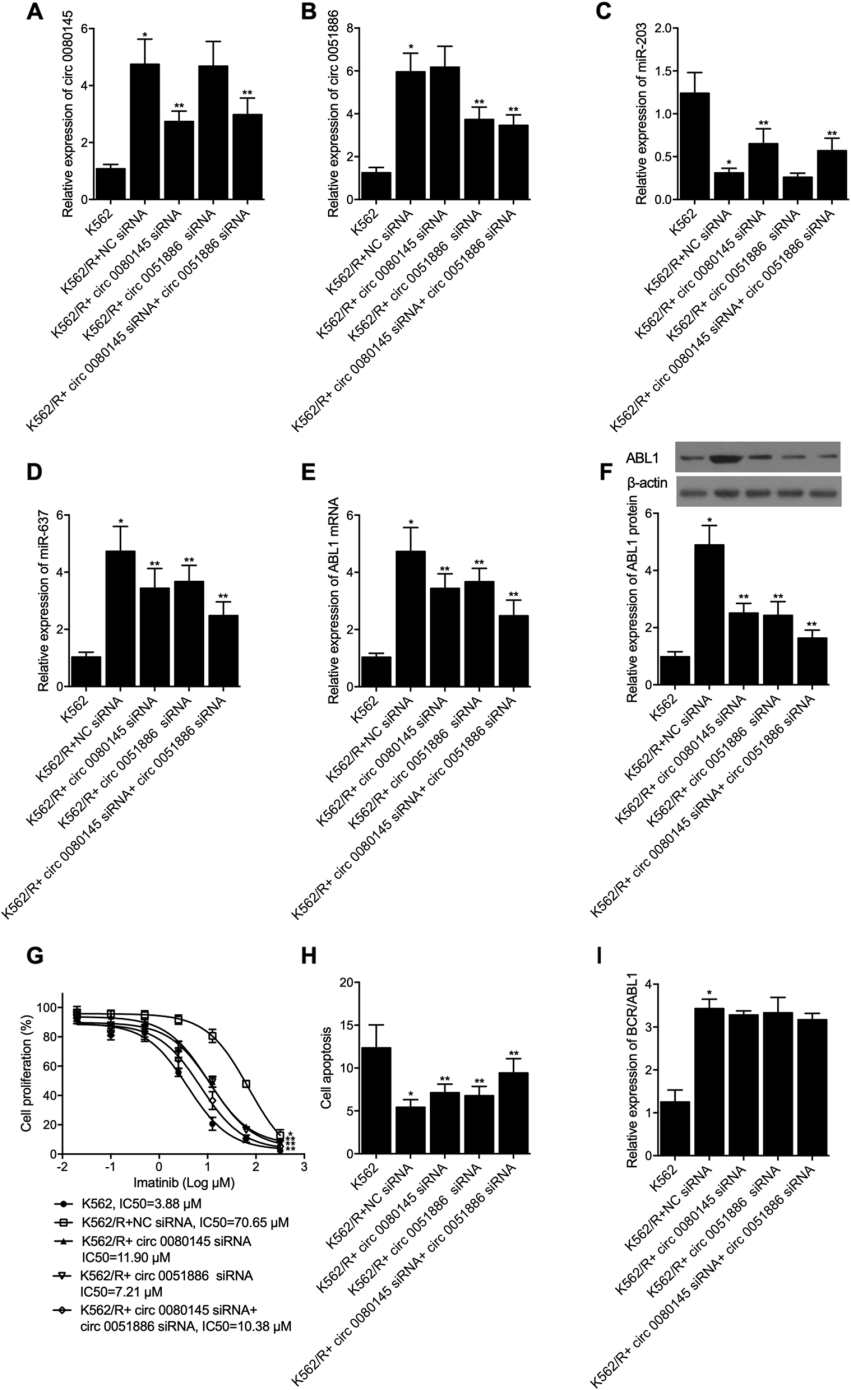
Expression of circ_0080145, circ 0051886, miR-203, miR-637, ABL1 mRNA/protein and BCR/ABL1 were compared between the K562 group, K562/R + NC siRNA group, K562/R + circ_0080145 siRNA group, K562/R + circ_0051886 siRNA group and K562/R + circ_0080145 siRNA + circ_0051886 siRNA group (*P value < 0.05 vs. K562 group; **P value < 0.05 vs. K562/R + NC siRNA group). **A** The expression of circ_0080145 was increased in K562/R cells, which was subsequently suppressed by the presence of circ_0080145 siRNA; **B** The expression of circ_0051886 was increased in K562/R cells, which was subsequently suppressed by the presence of circ_0051886 siRNA; **C** The expression of miR-203 was decreased in K562/R cells, which was subsequently promoted by the presence of circ_0080145 siRNA; **D** The expression of miR-637 was increased in K562/R cells, which was subsequently promoted by the presence of circ_0080145 siRNA and/or circ_0051886 siRNA; **E** The expression of ABL1 mRNA was increased in K562/R cells, which was subsequently promoted by the presence of circ_0080145 siRNA and/or circ_0051886 siRNA; **F** The expression of ABL1 protein was increased in K562/R cells, which was subsequently promoted by the presence of circ_0080145 siRNA and/or circ_0051886 siRNA; **G** Cell proliferation of K562/R cells was generally higher than K562 cells, and the presence of circ_0080145 siRNA and/or circ_0051886 siRNA reduced the decreased cell proliferation rate in K562/R cells to a certain degree; **H** The apoptosis rate of K562/R cells was lower than K562 cells, and the presence of circ_0080145 siRNA and/or circ_0051886 siRNA reduced the increased the cell apoptosis rate in K562/R cells to a certain degree; **I** The expression of BCR/ABL1 mRNA in were generally higher in K562/R cells, and the presence of circ_0080145 siRNA and circ_0051886 siRNA exhibited no effect upon the expression of BCR/ABL1 mRNA

The K562/R cells were transfected with NC siRNA (K562/R + NC siRNA group), circ_0080145 siRNA (K562/R + circ_0080145 siRNA group), circ_0051886 siRNA (K562/R + circ_0051886 siRNA group) and circ_0080145 siRNA, respectively, together with circ_0051886 siRNA (K562/R + circ_0080145 siRNA + circ_0051886 siRNA group). The expression of circ_0080145, circ 0051886, miR-203, miR-637 and ABL1 mRNA was observed. As shown in Fig. 5, the expression of circ_0080145 (Fig. 5A), circ_0051886 (Fig. 5B) and ABL1 mRNA (Fig. 5E) was elevated while the expression of miR-203 (Fig. 5C) and miR-637 (Fig. 5D) was downregulated in the K562/R + siRNA group compared with that in the K562 group. The transfection of circ_0080145 siRNA and circ_0051886 siRNA respectively led to the knockdown of circ_0080145 (Fig. 5A), circ_0051886 (Fig. 5B) and ABL1 mRNA (Fig. 5E) and protein (Fig. 5F) while upregulating the expression of miR-203 (Fig. 5C) and miR-637 (Fig. 5D). MTT assay was performed to observe the proliferation of K562 cells (Fig. 5G). The lowest proliferation rate was observed in the K562 cells and the highest proliferation rate was noted in the K562/R + NC siRNA group, and the transfection of circ_0080145 siRNA and circ_0051886 siRNA individually inhibited cell proliferation, and with the co-transfection of circ_0080145 siRNA and circ_0051886 siRNA exhibiting the most suppressing effect. On the contrary, FCM analysis upon cell apoptosis showed opposite tendency (Fig. 5H), with the K562/R + siRNA group showing the lowest apoptosis rate, which was reversed by the transfection of circ_0080145 siRNA and circ_0051886 siRNA. And the relative expression of BCR/ABL1 was higher in K562/R cells although circ_0080145 siRNA and circ_0051886 siRNA exhibited no effect upon the expression of BCR/ABL1 (Fig. 5I).

**Fig. 6:**
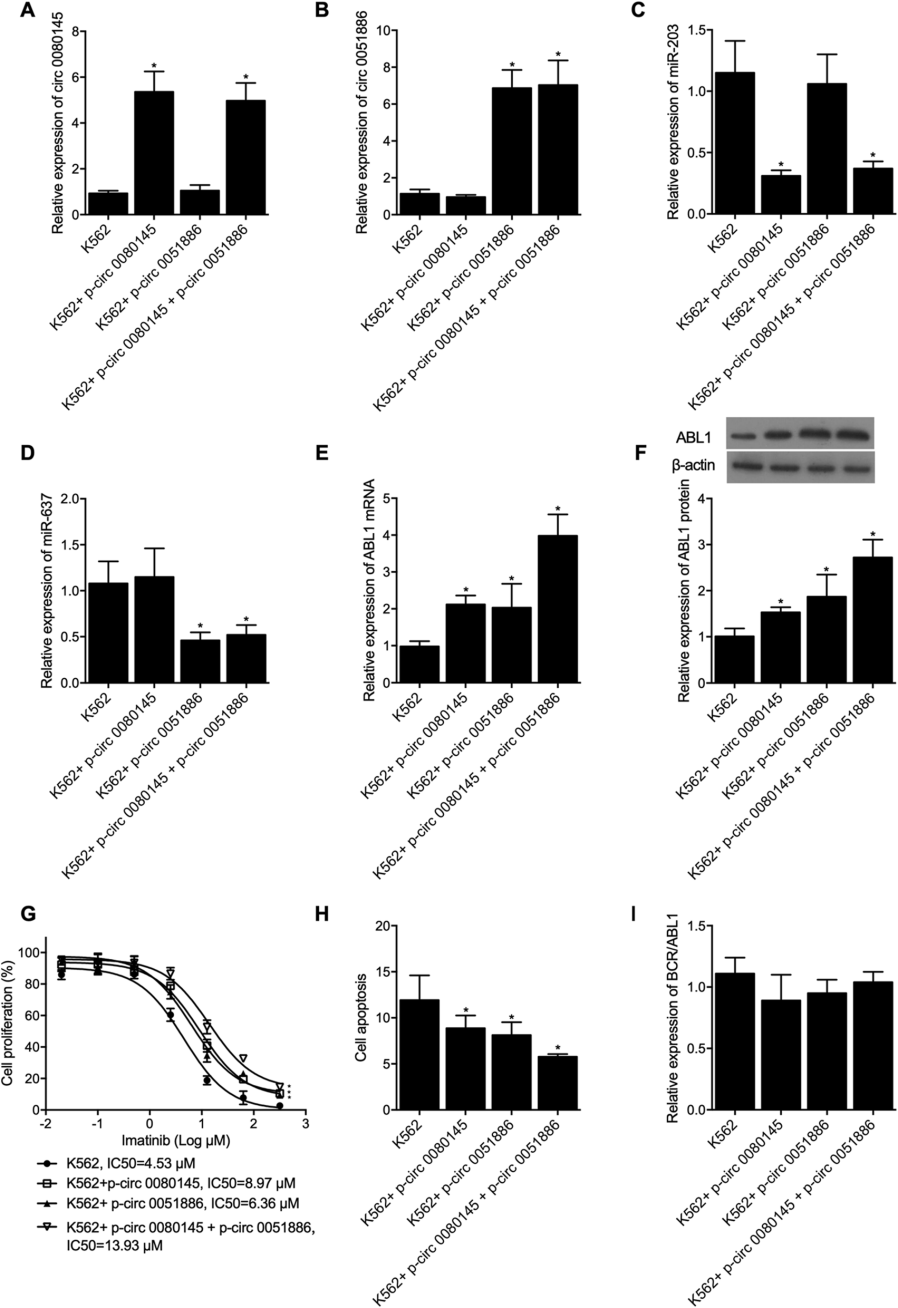
Expression of circ_0080145, circ 0051886, miR-203, miR-637 and ABL1 mRNA/protein and BCR/ABL1 between the K562 group, K562 + p-circ_0080145 group, K562 + circ_0051886 group and K562 + p-circ_0080145 + p-circ_0051886 group (*P value < 0.05 vs. K562 group). **A** The expression of circ_0080145 was increased by the presence of p-circ_0080145 in K562 cells; **B** The expression of circ_0051886 was increased by the presence of p-circ_0051886 in K562 cells; **C** The expression of miR-203 was decreased by the presence of p-circ_0080145 in K562 cells; **D** The expression of miR-637 was decreased by the presence of p-circ_0051886 in K562 cells; **E** The expression of ABL1 mRNA was increased by the presence of p-circ_0080145 and/or p-circ_0051886 in K562 cells; **F** The expression of ABL1 protein was increased by the presence of p-circ_0080145 and/or p-circ_0051886 in K562 cells; **G** The proliferation of K562 cells was promoted by the presence of p-circ_0080145 and/or p-circ 0051886; **H** The apoptosis rate of K562 cells was suppressed by the presence of p-circ_0080145 and/or p-circ 0051886; **I** The expression of BCR/ABL1 mRNA was not influenced by the presence of p-circ_0080145 and/or p-circ_0051886 in K562 cells

Moreover, K562 cells were transfected with plasmids expressing circ_0080145 (K562 + p-circ_0080145 group) and circ_0051886 (K562 + circ_0051886 group), individually, or the co-transfection of circ_0080145 and circ_0051886 (K562 + p-circ_0080145 + p-circ_0051886 group). The successful transfection was validated by observation of upregulated expression of circ_0080145 (Fig. 6A) and circ_0051886 (Fig. 6B). The over-expression of circ_0080145 reduced the expression of miR-203 (Fig. 6C), and the over-expression of circ_0051886 reduced the expression of miR-637 (Fig. 6D). Accordingly, the expression of ABL1 mRNA (Fig. 6E) and protein (Fig. 6F) was downregulated by the over-expression of circ_0080145 or circ 0051886, while the expression of ABL1 was the highest when both circ_0080145 and circ_0051886 were upregulated. Also, cell proliferation rate was promoted (Fig. 6G) but cell apoptosis was suppressed (Fig. 6H) by the over-expression of circ_0080145 and circ 0051886, with the co-transfection of circ_0080145 and circ_0051886 exhibiting the most promoting effect. Moreover, the over-expression of circ_0080145 and/or circ_0051886 exhibited no effect upon the expression of BCR/ABL1 (Fig. 6I).

## Discussion

CML is a type of myeloproliferative disorder caused by the malignant transformation of hematopoietic stem cells (Fialkow et al. 1977). On the molecular level, the outcome of the reciprocal translocation t (9;22) (q34q11) in CML is the formation of an oncogene termed BCR/ABL, which encodes a chimeric protein, BCR-ABL, with constitutive tyrosine kinase activity (Sun et al. 2013). As a major first-line treatment of CML, IM shows excellent performance in CML treatment but its chemoresistance remains a key issue (Sun et al. 2013; Ren 2005). Chemoresistance usually develops via subclones carrying genetic mutations allowing the host subclones to escape the cytotoxicity of IM, while other molecular mechanism is also indicated (Zhou and Xu 2015). In this study, we recruited CML subjects treated with imatinib. The participants were grouped based on their response to the imatinib treatment as responders and non-responders. We evaluated the expression of some candidate circRNAs in samples collected from both groups and we found that as compared with that in the responder group, the expression of circ_0080145, circ 0134501 and circ_0051886 was all significantly increased, while the expression of circ 0134491 was downregulated in the non-responders. Meanwhile, the expression of miR-203 and miR-637 in the responder group was both evidently downregulated. Those results further confirmed the microarray data reported previously (Liu et al. 2018) and provided us clue for further exploration of the molecular pathways.

It has been repeatedly reported that circRNAs might interact with miRNAs to sponge the production and block the function of certain miRNAs or mRNAs (Dong et al. 2017; Li et al. 2017). In a study investigating the top twenty circRNAs aberrantly expressed in leukemia, hsa _circ_0080145, hsa_circ_0024002, as well as hsa_circ_0037781 were found to be the highest number of miRNA targets, including miRNAs associated with leukemia such as miR-29b, miR-181a as well as miR-16. In particular, miR-16 expression was upregulated in peripheral lymphoid tissues, while miR-181a expression could reduce the level of vascular inflammation in macrophages, indicating that these circRNAs might participate in CML pathogenesis (Musolino et al. 2018; Du et al. 2018). The results that certain miRNAs such as miR-203 regulated ABL1 expression suggest that these miRNAs may be beneficial to the patients of ALL or CML carrying the fusion genes of NUP214-ABL1 or BCR-ABL1 (Bueno et al. 2008; Furuta et al. 2010). Just recently, the deregulation of hsa-miR-203 caused by aberrant methylation was found in many patients of CML as well as hepatocellular carcinoma, suggesting that the methylation and silencing of miR-203 can benefit the growth of tumor cells (Furuta et al. 2010; Bueno et al. 2016). In this study, we established K562 cells that were resistant to the killing effect of imatinib and evaluated the expression of circRNAs and miRNAs that were differentially expressed in the two cell groups. The results showed that the expression of circ_0080145, circ 0134491 and circ_0051886 was upregulated in the K562/R group, while the expression of circ 0134501 was downregulated in the K562/R group. Furthermore, by using bioinformatic tools and luciferase assay, we predicted that circ_0080145 and circ_0051886 could target miR-203 and miR-637 respectively, while both miR-203 and miR-637 were proven to target ABL1 mRNA, indicating the establishment of two potential signaling pathways, circ_0080145/miR-203/ABL1 and circ 0051886/miR-637/ABL1. Comparison of data in Figs. 1 and 2, the trend of changes of circ_0080145 and circ_0051886 was consistent with each other, while some other circRNAs such as circ_0134501 and circ_0134491 were opposite. The data from Fig. 1 was obtained from multiple patients, either sensitive or resistant, and the data form Fig. 2 was obtained from a cell line and its resistant derivatives which could be deemed as one single individual. The reason that we used both primary cells as well as culture cells is that, on one hand, the RNA expression is variable and could be transient, and the individual difference of the primary cells or the patients will influence the accuracy of the results. On the other hand, K562 cells were more easily to culture with better growth conditions. And Also, K562, as a culture cell line derived from CML patient, could also be deemed as an individual, but well established, easily maintained and ready for various assays. Therefore, we only selected the ones that are consistent between the human subjects and cell line for further functional analysis. In this study, we found that the knockdown of circ_0080145 and circ_0051886 reduced the expression of ABL1 mRNA and BCR/ABL1 protein. It was noteworthy that the co-transfection of circ_0080145 siRNA and circ_0051886 siRNA most significantly suppressed the proliferation while promoting the apoptosis of K562/R cells. On the contrary, the over-expression of circ_0080145 and circ_0051886 respectively reduced the expression of miR-203 and miR-637. The expression of ABL1 and BCR/ABL1 proteins was most upregulated with the co-transfection of circ_0080145 and circ 0051886.

IM resistance has raised great attention while prompting interest in the development of other treatment strategies, so as to achieve a cure without the requirement for the transplantation of stem cells (Hochhaus et al. 2002; Druker et al. 2006; Lewandowski et al. 2009). The data of this study explore a novel molecular mechanism other than point mutations in ABL and provided us another strategy to improve the sensitivity to IM especially in those subjects with IM resistance.

## Conclusion

This study established the signaling pathways of circ_0080145/miR-203/ABL1 and circ 0051886/miR-637/ABL1. The deregulation of circ_0080145 and circ_0051886 is, at least partially, responsible for the development of IM chemoresistance in CML by regulating expression of ABL1 via modulating expression of miR-203 and miR-637.

## Data Availability

The data that support the findings of this study are available from the corresponding author upon reasonable request.

## Abbreviations

CML: Chronic myeloid leukemia
IM: Imatinib
